# Pseudogenization of the chaperonin system in ‘Candidatus Phytoplasma pruni’ revealed by genome sequencing and comparative genomics

**DOI:** 10.1099/mgen.0.001748

**Published:** 2026-06-08

**Authors:** Thierry Alexandre Pellegrinetti, Christine Hammond, Edel Pérez-López, Kevin Muirhead, Harvinder Bennypaul, Daniel Sanderson, Tim J. Dumonceaux

**Affiliations:** 1 Département de phytologie, Faculté des sciences de l'agriculture et de l'alimentation, Université Laval, Québec City, Canada; 2 Agriculture and Agri-Food Canada Saskatoon Research and Development Centre Saskatoon SK S7N 0X2, Saskatoon, Canada; 3 Department of Biochemistry and Molecular Biology, University of Calgary, Calgary, Canada; 4 Center for Plant Health, Canadian Food Inspection Agency, North Saanich, BC, V8L 1H3, Canada

**Keywords:** *Acholeplasma*, evolution, genome reduction, phytoplasma, pseudogenes

## Abstract

GroE is a chaperonin folding system consisting of GroEL (Cpn60, a 60 kDa chaperonin), and the smaller co-chaperonin GroES (Cpn10). Many ‘client’ proteins require GroE to fold properly, including several that are essential for cell viability. GroE is found in nearly all bacteria and eukaryotes. *Mollicutes* are the only micro-organisms that lack GroE in almost all cases. Only two clades of *Mollicutes* have retained the ancestral GroE system, or perhaps reacquired one; these exceptions include the family *Acholeplasmataceae*, consisting of the genera *Acholeplasma* and ‘*Candidatus* Phytoplasma’. The role of GroEL in these unique *Mollicutes* is a source of speculation, given how many non-canonical ‘moonlighting’ roles have been ascribed to this protein. GroEL has been suggested to play a role in pathogenesis in plant and animal pathogenic *Mollicutes* by binding to host cells and facilitating invasion. However, in one further layer of exception, the phytopathogenic taxon ‘*Candidatus* Phytoplasma pruni’ (ribosomal group 16SrIII) was reported in 2012 to lack a GroE system. This study confirms the lack of a functional GroE system in 16SrIII by providing two new, high-quality, non-fragmented genome assemblies, as well as a thorough survey of other 16SrIII genomes for genes encoding GroEL/GroES, including those that may not resemble phytoplasma GroEL (i.e. acquired by horizontal gene transfer, HGT). We discuss the implications of a clearly phytopathogenic, invasive group of *Mollicutes* that nevertheless lacks GroE, in light of the presumed role of GroEL for this species. We determined that multiple genomes of 16SrIII contain short, non-functional *groEL* pseudogenes, while most of the reported genomes lack any semblance of a GroE system. Examination of the new assemblies allowed us to rule out HGT as a means of GroE acquisition.

**Impact Statement**

*Mollicutes* depend on host cells to replicate and survive, and many cause disease in animals and plants. Their obligate intracellular lifestyle, combined with extensive genome reduction, has long complicated efforts to understand the molecular mechanisms underlying infection. Among the systems commonly lost during this reductive evolution is the GroE chaperonin complex (GroES–GroEL), best known for its essential role in protein folding but also associated with diverse pathogenic functions in other bacteria. Using genome sequencing and comparative genomics, we determined that phytoplasmas within the 16SrIII group have independently lost a functional GroE system. Approximately half of the analysed genomes retain only a vestige of a fragmented *groEL* pseudogene, whereas the remainder show a complete absence of GroE. This pattern indicates an ancient gene loss event and suggests that GroEL is unlikely to contribute to virulence in this lineage. Instead, these phytoplasmas may rely more heavily on host-derived chaperonin or alternative folding pathways to maintain proteostasis. By revealing differential retention of GroE among phytoplasma lineages, this study provides new insight into the evolutionary pressures shaping their minimalist genomes. It highlights gene loss as a dynamic and lineage-specific process and raises broader questions about how intracellular bacteria adapt, maintain protein function and evolve pathogenicity in the absence of canonical cellular machinery.

## Data Summary

The authors confirm that all supporting data, code and protocols have been provided within the article or through supplementary data files. Supplementary files are provided via Figshare (https://doi.org/10.6084/m9.figshare.32145184).

## Introduction

*Mollicutes*, so named because of their lack of a cell wall (Latin *mollis*, soft, + *cutis*, skin), are a major group within the Gram-positive phylum *Bacillota* (formerly *Firmicutes*). This class of bacteria is believed to have evolved around 600 million years ago [1] through a reductive process whereby the micro-organisms reduced the metabolic capacities and structural features encoded on their genomes as they adopted an obligately intracellular lifestyle. Extant *Mollicutes* are universally dependent on eukaryotic host cells to complement their limited metabolic functions, although a few, notably mycoplasmas, can be cultured in complex artificial medium and are a common contaminant of eukaryotic cell cultures [2]. *Mollicutes* are commonly associated with pathogenesis in plants (*Spiroplasma*, ‘*Candidatus* Phytoplasma’) [3,4] and animals (*Mycoplasma*) [5]. Due to their reduced genomes, *Mollicutes* are among the bacteria with the smallest genome size – *Mycoplasma genitalium* possesses one of the smallest known bacterial genomes at 580 kbp [6]. Other unique features of Mollicute genomes include a low G+C content, and the use of the codon UGA for tryptophan rather than a stop codon as in other bacteria [7]. The single exception to the latter characteristic is the family *Acholeplasmataceae*, which consists of the two genera *Acholeplasma* and ‘*Candidatus* Phytoplasma’. Since these taxa use UGA as a stop codon, which is characteristic of non-Mollicute bacteria, they are presumed to be more closely related to the ancestral taxon [1,8].

The protein chaperonin system (GroE) consists of the two proteins GroEL (synonym Cpn60) and GroES (synonym Cpn10) [9]. This system is canonically involved in the prevention of aggregate formation and proper folding of cellular proteins as a part of the basic protein biosynthetic machinery, and for this reason, these two genes are anticipated to be present in all prokaryotic and eukaryotic cells [10]. GroE ‘client proteins’ include several that are essential for cell viability, so that a loss of this system is thought to result in a cell that is nonviable [11]. *Mollicutes*, however, are also unique in being the only known cells that generally lack a GroE system, although it is present in a subset of these micro-organisms [12]. Because the GroE system is an ancestral property (i.e. it is present in all non-*Mollicute* bacteria), it is thought that *Mollicute*s lost these genes during genome reduction [11,12]. The retention, or re-acquisition, of GroE is polyphyletic, with two groups of *Mollicutes* featuring these genes: a smaller clade consisting of *Mycoplasma gallisepticum*, *M. genitalium* and *Mycoplasma pneumoniae*, and a much larger group, including nearly all of the acholeplasmas and phytoplasmas [11]. In addition, there are at least three clear instances of the acquisition of *groEL* by horizontal gene transfer (HGT) in *Mollicutes*: the gene of *Mycoplasma penetrans* is more closely related to that of *Helicobacter pylori* than other mycoplasmas [12], and two species of *Spiroplasma*, *Spiroplasma turonicium* and *Spiroplasma kunkelii*, appear to have acquired *groEL* genes by HGT [11].

These observations have led to speculation regarding the role of these proteins within these intracellular micro-organisms, particularly since a very wide array of non-canonical ‘moonlighting’ roles has been ascribed to GroEL in prokaryotic and eukaryotic cells [13]. Notably, Clark and Tillier [12] proposed that, among *Mollicutes* that encode GroEL in their genomes, the protein may play a role in pathogenesis by acting as an adhesin/invasin, suggesting that the re-acquisition or retention of the GroE chaperonin system may be associated with virulence. This notion is supported by a wide variety of observations that non-Mollicute bacteria can localize GroEL to the cell surface or even secrete the protein into the extracellular space, clearly indicating a non-canonical, virulence-associated role for the protein in certain bacteria [14,16]. These considerations provide strong reasons to consider carefully the role of GroEL and the GroE system in pathogenic *Mollicutes* that have retained or re-acquired these genes.

Phytoplasmas, plant pathogenic micro-organisms that are transferred between host plants through an insect vector [17], are among the two groups of *Mollicutes* that are typically considered to have retained or re-acquired a GroE system [11]. However, an exception to this was noted when the genomes of four members of the ribosomal group 16SrIII, classified as ‘*Candidatus* Phytoplasma pruni’ were sequenced, revealing a lack of genes annotated as *groES* or *groEL* in these taxa [18]. However, all of these genomes are highly fragmented, with at least 150 contigs present in each. Phytoplasma genomes have proven to be difficult to sequence prior to the introduction of long-read sequencing technologies. Despite technological developments for sample preparation, including antibody-based enrichment [19], many phytoplasma genomes deposited in public repositories remain fragmented and incomplete. This hinders the search for *groEL* and *groES* genes in phytoplasma genomes and can mask the possibility of gene acquisition by HGT. Thus, the reported lack of a GroE system in this singular group of phytoplasmas remains an unresolved question.

We address this issue by examining all reported genomes of ‘*Candidatus* Phytoplasma pruni’ for the presence of *groEL*/*groES* genes or pseudogenes and provide two new high-quality genome assemblies for 16SrIII phytoplasmas in subgroups A and F. We report the pseudogenization of *groEL* and a complete lack of *groES* in four groups of these phytoplasmas and have confirmed through analysis of all reported high-quality phytoplasma genomes that the 16SrIII group constitutes the only phytoplasmas that truly lack a chaperonin system. This observation supports the lack of a moonlighting role for GroEL in host cell attachment and invasion and, supported by our findings, instead suggests that the protein primarily fulfils its canonical function of folding client proteins in phytoplasmas.

## Methods

### Strain sources and sampling

**Fig. 1.**
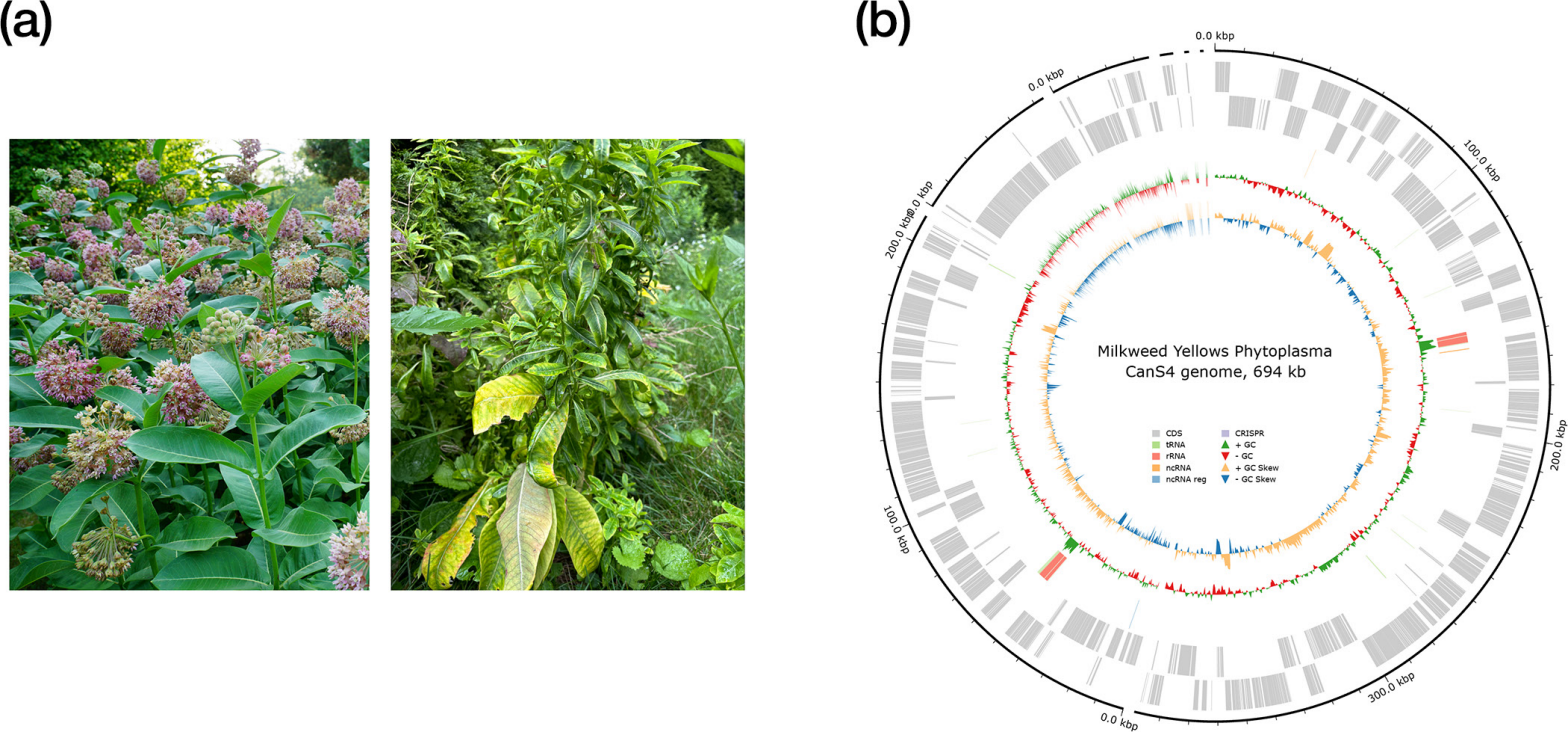
(a) Healthy (image purchased from shutterstock.com) and symptomatic milkweed (observed at the Canadensis Botanical Garden, Ottawa, Ontario, Canada). (b) Genome map of MYp-CanS4.

*Syringa* (lilac) infected with ‘*Ca*. P. pruni’ (16SrIII) strain 2A1 was maintained at the Centre for Plant Health, Canadian Food Inspection Agency, North Saanich, British Columbia, Canada. Symptomatic milkweed (*Asclepias* sp.) was observed at the Canadensis Botanical Garden, Central Experimental Farm of Agriculture and Agri-Food Canada, Ottawa, Ontario, Canada (45° 23′ 03.8″ N 75° 42′ 19.3″ W) (Fig. 1). The sample taken from one of these plants (S4) is called MYp-Canadensis (Milkweed Yellows phytoplasma-Canadensis).

### DNA extraction, PCR and sequencing

For both plant types, leaf tissue (midrib and petiole) was cut from infected leaves and homogenized in liquid nitrogen using a mortar and pestle that was pre-treated with DNAaway (Thermo Fisher). DNA was extracted from the plant material using a Wizard High Molecular Weight DNA extraction kit (Promega). DNA yield was determined using a Qubit fluorometer (Thermo Fisher), and molecular weight was assessed using agarose gel electrophoresis.

Phytoplasma infection of the milkweed tissue was confirmed using a qPCR assay targeting 16S rRNA genes as described [20]. The phytoplasma was typed using nested PCR amplification of 16S genes (F2nR2) [21,22], and cloned amplicons were sequenced by Sanger (Eurofins). The Restriction Fragment Length Polymorphism (RFLP) type was determined for six clones using the iPhyClassifier [23].

Bacterial DNA was enriched from the leaf DNA extract using a NEBNext Microbiome Enrichment Kit (New England Biolabs) as previously described [24]. Both short-read Illumina and long-read Oxford Nanopore Technologies (ONT; nanopore) were used for genome sequencing. For Illumina, 0.5 µg of enriched DNA was used as the input material for each DNA library preparation. The sequencing libraries were generated using a NEBnext UltraII FS DNA lib prep kit E7805 >100 ng, according to the manufacturer’s instructions, with the following specifications: 10 min fragmentation, size selection using 30 µl/10 µl beads for 200–475 bp, 5 cycles of amplification and use of dual index barcodes using NEBnext multiplex oligos for Illumina (NEB, USA). The DNA libraries were sequenced on an Illumina NovaSeq 6000 platform (Illumina, San Diego, USA), and 150 bp paired-end reads were generated. For ONT sequencing, 0.12 µg for bacterial-enriched lilac and 0.525 µg for bacterial-enriched milkweed DNA were used as the input material for the library preparations. The sequencing library was prepared using an ONT Ligation Sequencing Kit (LSK-SQK114), followed by sequencing on a MinION Mk1C device (ONT, Oxford, UK).

### Bioinformatics

Illumina reads were quality-trimmed using Trimmomatic v0.39 [25], by removing bases from the beginning of reads with a quality score below 3, from the end of reads with a quality score below 20, and cutting any reads with a quality score below 15 over a sliding window of 4 bases. Reads shorter than 36 bases after trimming were removed. The Illumina reads from the lilac-2A1 sample were then mapped (without merging, to minimize read loss) using a reference database prepared from ‘*Ca*. P. pruni’ strain PR2021 (GCA_029746895) with Bowtie2 v2.4.1 [26]. The MYp-Canadensis Illumina reads were mapped using BWA v0.7.17-r1188 [27] with a reference database prepared from the previously reported milkweed yellows genome (GCA_000309485). Nanopore reads were not mapped but were filtered to exclude reads under 1,000 bp and retain the top 90% of reads based on quality scores using Filtlong v0.2.1 (https://github.com/rrwick/Filtlong). Illumina and nanopore reads were co-assembled using Unicycler v0.5.0 [28]. Assemblies were examined for the possible presence of plasmids using Plasmer v0.1 20220816 [29], and assembly completeness was assessed using Busco v5.8.2 [30] with the default *Mollicutes* reference database (mollicutes_odb10). Completeness and contamination of the genomes were also examined using CheckM2 v1.1.0 [31]. Assembled genomes were annotated locally using Bakta v1.11.0 [32], as well as by NCBI using PGAP [33].

To examine the sequencing data for evidence of HGT, a separate assembly was prepared for the MYp-Canadensis sample by removing host reads (including chromosomes, mitochondria and chloroplast) using BMTagger v3.101 [34]. Chromosomal sequences for *Asclepias syriaca* were downloaded from milkweedbase.org, while chloroplast (NC_022432.1) and mitochondrial (NC_022796.1) genomes were downloaded from NCBI. Nanopore reads were filtered to exclude those under 1,000 bp and retain the top 90% by quality score using Filtlong v0.2.1, then mapped to remove host reads using Minimap2 v2.28 [35]. These reads were then used to generate a Unicycler assembly from all non-host reads. Any assembly fragments that contained a *groEL* gene (identified by blast) were typed at cpnDB (https://research-groups.usask.ca/hilllab/cpndb.php), and the sequences flanking *groEL* were analysed by blast at NCBI to determine if the genomic context of the identified *groEL* gene provided the same taxonomic identification.

### Examination of previously reported phytoplasma genomes

To retrieve all phytoplasma genomes from GenBank, the NCBI Genomes database (https://www.ncbi.nlm.nih.gov/datasets/genome/) was queried using the search term ‘phytoplasma’. This search (1 January 2025) retrieved 271 genomes, from which a single example of each unique taxid was selected. In cases where multiple taxid were represented, the genome with the highest completeness score reported was selected. Genomes with a reported CheckM completeness score below 90% were excluded from analysis. This resulted in 65 phytoplasma genomes in the final dataset (Table S2, available in the online Supplementary Material). Genomes corresponding specifically to group 16SrIII were downloaded from NCBI using the information provided by Fernández *et al*. [36] (Table 1). Genes annotated as *groEL*, *groES* and 16S rRNA were selected from each genome outside of group 16SrIII. In addition, taxonomic markers *secY*, *secA*, *tuf* and *nusA* [37] were retrieved from peanut witches’ broom phytoplasma (PnWB; GCA_000364425.1) and from selected 16SrIII genomes.

**Table 1.** ‘*Candidatus* Phytoplasma pruni’ group 16SrIII genomes included in the analysis reported in this work. This table is based on and modified from Fernández *et al.* [36]

Phytoplasma	Strain	16SrIII-subgroup	Genome level (fragments – completeness)	Host	Location	Accession	Genome size (kb)	Reference
*Cicuta witches’ broom*	CicWB	16SrIII-J	Contig (16–96.7%)	*Conium maculatum*	Argentina	GCA_035853675.1	758	[36]
*Phytoplasma Vc33*	Vc33	16SrIII-J	Contig (36–87.4%)	*Catharanthus roseus*	Chile	GCA_001623385.2	687	[57]
*China tree decline*	ChTDIII	16SrIII-B	Contig (67–97.4%)	*Melia azedarach*	Argentina	GCA_013391955.1	791	[58]
*Italian clover phyllody*	MA	16SrIII-B	Contig (197–94.0%)	*C. roseus*	Italy	GCA_000300695.1	597	[18]
*Poinsettia branch-inducing*	JR	16SrIII-A	Contig (185–96.0%)	*Euphorbia pulcherrima*	USA	GCA_000309465.1	631	[18]
*Milkweed yellows phytoplasma*	MW	16SrIII-F	Contig (158–94.0%)	*C. roseus*	Canada	GCA_000309485	584	[18]
*Vaccinium witches’ broom*	VAC	16SrIII-F	Contig (272–96.7%)	*Vaccinium myrtillus*	Italy	GCA_000309405.1	648	[18]
‘*Candidatus* Phytoplasma pruni’	PR2021	16SrIII-A	Chromosome (1–97.4%)	*E. pulcherrima*	Taiwan	GCA_029746895.1	710	[59]
‘*Candidatus* Phytoplasma pruni’	CX	16SrIII-A	Contig (46–93.4%)	*C. roseus*	USA	GCA_001277135.1	599	[60]
Lettuce witches’ broom phytoplasma	LWB	16SrIII-X	Chromosome (1–96.7%)	*Lactuca sativa*	Argentina	GCA_051397825.1	659	[61]
*Milkweed yellows phytoplasma*	MYp-CanS4	16SrIII-F	Contig (7–97.4%)	*A. syriaca*	Canada	GCA_050286905.1	694	This work
‘*Candidatus* Phytoplasma pruni’	2A1	16SrIII-A	Contig (2–97.4%)	*Lilac*	Canada	GCA_033391615.1	625	This work

### Phylogenetic analysis

Phylogenetic trees were generated to evaluate both taxonomic placement and gene-level relationships among phytoplasma genomes. The 16S rRNA genes were extracted from all genomes using the annotation done in Bakta and aligned to representative sequences of each phytoplasma group. Maximum likelihood phylogenetic reconstruction was performed using IQ-TREE2 [38] with automatic model selection and branch support estimated by 1,000 ultrafast bootstrap replicates (UFBoot2). A second phylogenetic analysis was performed using the *groEL* gene sequences, also obtained from Bakta annotations. The *groEL* sequences were aligned, and a maximum likelihood tree was constructed with IQ-TREE2 using the same parameters and ultrafast bootstrap support estimation. The topology was visualized and annotated using the Interactive Tree of Life (iTOL) [39] for direct comparison with the 16S-based tree. The resulting trees were visualized and annotated using iTOL.

### Examination of 16SrIII genomes for sequences related to *groEL* and *groES*

Each of the downloaded genomes was used to prepare a local blast database. The 16SrIII genome 2A1 was queried using tBLASTn, and a GroEL amino acid sequence from ‘*Ca*. P. asteris’ (strain AYWB; cpnDB ID b8392; 16SrI) as input. This retrieved a short DNA sequence (165 bp) that encoded a predicted protein of 55 amino acids with a BLASTp match to phytoplasma GroEL. This sequence was then used to query all other 16SrIII genomes using BLASTn. The predicted amino acid sequences of each of the regions with significant matches (based on low e values over more than 20 nucleotides) were compared to the GenBank database using BLASTp, and those with a strong match to GroEL were considered to be potential pseudogenes. A similar approach was used for *groES*.

### Structural analysis of GroEL proteins encoded by intact genes and pseudogenes

Structural models for intact GroEL (16SrII, 16SrIX) and pseudogene-predicted fragments from lettuce WB (16SrIII) were generated using AlphaFold 3 [40]. PDB files were downloaded, and structural modelling and alignments were performed with UCSF ChimeraX 1.10.1 [41].

### Selection pressure analysis

To test for relaxed selection in fragmented *groEL* sequences, we performed comparative dN/dS analysis using RELAX v4.5 [42] via HyPhy v2.5. Intact *groEL* sequences (*n*=133) from diverse phytoplasma groups were extracted from GenBank genomes using tBLASTn. Fragmented *groEL* remnants from the six 16SrIII genomes that were found to contain *groEL* pseudogenes (Table 2) were identified using more relaxed criteria (e-value ≤10⁻⁵, length ≥100 bp) compared to their initial identifications. This was done to maximize fragment recovery for analysis of the evolution and decay of the GroEL-encoding region in these genomes and resulted in the identification of 13 *groEL* gene fragments in the 6 genomes.

**Table 2.** Amino acid lengths and genome coordinates of *groEL* pseudogenes and genes immediately upstream in 16SrII and 16SrIII

			*groEL* (pseudo)gene			*evbG* orthologue				*evbH* orthologue		
**Phytoplasma**	**Strain**	**16SrIII-subgroup**	bp*	AA*	**Coordinates (contig:bases)**	**bp**	**AA**	**Coordinates (contig:bases)**	**Intergenic distance, bp†**	**bp**	**AA**	**Coordinates (contig:bases)**
Italian clover phyllody	MA	16SrIII-B	153	50	33 : 3851–4003	1,728	576	33 : 1856–3586	266	1,782	593	33 : 78–1859
Poinsettia branch-inducing	JR	16SrIII-A	186	62	171 : 1963–2148	1,703*	566*	5 : 13448–15150	ND‡	1,782	593	5 : 11670–13451
‘*Candidatus* Phytoplasma pruni’	PR2021	16SrIII-A	186	62	686562–686747	1,728	576	684481–686211	352	1,782	593	682703–684484
‘*Candidatus* Phytoplasma pruni’	CX	16SrIII-A	165	55	8 : 22632–22796	1,728	576	8 : 20474–22204	429	1,782	593	8 : 18696–20477
‘*Candidatus* Phytoplasma pruni’	2A1	16SrIII-A	165	55	1 : 56157–56321	1,728	576	1 : 56749–58479	159	1,782	593	1 : 58476–60257
Lettuce witches’ broom phytoplasma	LWB	16SrIII-X	787	180/87§	1 : 234271–235038	1,734	578	1 : 235221–236954	184	1,779	593	1 : 236954–238732
Peanut witches' broom phytoplasma	PnWB	16SrII-A	1,638	546	9 : 28499–30136	1,731	577	9 : 26551–28284	216	1,842	613	9 : 24713–26554

*bp, base pairs; AA, amino acids.

†*groEL*-evbG intergenic distance in base pairs.

‡Minus strand – contig 5 length is 15,150 bp. Therefore, the junction of *evbG* and *groEL* pseudogene is disrupted, and intergenic distance cannot be calculated.

§Two polypeptides are annotated as *groEL/cpn60* on this fragment, with frameshifts and stop codons in the sequence. The entire region is considered a pseudogene.

Sequences were classified based on length, reading frame integrity and presence of internal stop codons. Sequences maintaining minimal structural requirements (length ≥100 bp, ≤2 internal stop codons) were aligned at the amino acid level using MAFFT v7.475 (--auto option), followed by codon-aware back-translation. A maximum likelihood phylogenetic tree was constructed using IQ-TREE v2.0 (ModelFinder+1,000 ultrafast bootstrap replicates).

For RELAX analysis, fragmented sequences were designated as 'Test' branches and intact sequences as 'Reference' branches. The analysis estimates a relaxation parameter (k), where k<1 indicates relaxed selection, k=1 indicates unchanged selection, and k>1 indicates intensified selection. Statistical significance was assessed via a likelihood ratio test comparing alternative (k≠1) versus null (k=1) models.

Due to advanced pseudogenization (mean fragment length 204 bp vs 1,437 bp intact), only 1 of 13 fragmented sequences met the minimum requirements for codon-level analysis, limiting statistical power for detecting selection relaxation.

### Examination of the gene neighbourhood of *groEL* in group 16SrII and 16SrIII phytoplasmas

The annotated genomes of ‘*Ca*. P. pruni’ strain CX (Ga0100078) and ‘*Ca*. P. aurantifolia’ strain PnWB (Ga0248296) at the Genomes Online Database (https://gold.jgi.doe.gov/) were used to examine the gene neighbourhood of the intact GroE system in PnWB compared to the putative pseudogene identified in strains CX and 2A1. Genes immediately downstream of the annotated *groEL* gene or gene fragment from each strain were downloaded and used to identify the corresponding genes in each of the group III phytoplasmas that were suspected to harbour *groEL* pseudogenes. The locations and annotations of the genes corresponding to the immediate gene neighbours of the GroE system in PnWB were noted in each strain, and intergenic distances were calculated. Both gene and predicted amino acid sequences of these genes were downloaded and used for sequence similarity determination.

The gene regions containing *groEL* genes or putative pseudogenes were selected from each of the 16SrIII genomes, and from related strains from groups 16SrII (PnWB strain NTU2011; NZ_AMWZ01000009.1) and 16SrIX (‘*Ca*. P. phoenicium’ strain SA213; NZ_JPSQ01000038.1). The genomic regions were annotated using Prokka v 1.14.6 [43] and aligned and visualized using clinker v0.0.31 [44]. A comparative pangenome analysis was also performed using Anvi’o v.8 [45], with functional annotation based on COG categories. The GroE cluster was examined across the pangenome to assess gene presence, absence and fragmentation patterns indicative of pseudogene formation.

## Results and discussion

### Genome sequencing of ‘*Ca*. P. pruni’ strains 2A1 and MYp-Canadensis S4

Lilac (*Syringa* sp.) infected with 16SrIII strain 2A1 was experimentally maintained in greenhouses in North Saanich, British Columbia, Canada. In total, 57,198 nanopore reads and 1.5M Illumina reads (host-depleted) were obtained from the infected lilac sample. The genome sequence of strain 2A1 was 625 kb in length (Table 1), with a G+C content of 27.32 mol%. The estimated coverage of the genome was ~600× (800× for nanopore and 364× for Illumina, considering read length and number). The genome consisted of two scaffolds with lengths of 336 and 289 kb. A total of 594 protein-coding genes were predicted in the genome, 437 of which had a function prediction. In addition, the genome contained two copies of 16S rRNA, as is typical for phytoplasmas [46]. Both genes were typed at the iPhyClassifier as 16SrIII-A (F=1.0), indicating a lack of the 16S rRNA gene heterogeneity that has been observed in some phytoplasmas in the 16SrIII group [47].

Milkweed (*Asclepias syriaca*) was observed at the Canadensis Botanical Garden with symptoms of chlorosis, stunting and phyllody (Fig. 1a). Symptomatic plants were confirmed to be infected by phytoplasma using qPCR targeting 16S rRNA genes. The 16S rRNA genes were amplified by PCR, sequenced and examined using RFLP analysis. This showed that strain MYp-Canadensis (S4) was typed as 16SrIII-F (F=1.0), again with no evidence of gene heterogeneity. This is consistent with previous RFLP typing results of milkweed yellows phytoplasma [18]. The geographic location of the MYp detected in this study is consistent with the original report of MYp in Tichbourne, Ontario, in the early 1990s [48], indicating that this phytoplasma has been circulating in Eastern Canada for over three decades.

A total of 2.8M Illumina reads and 31,281 nanopore reads (quality-filtered, host-depleted) were obtained from the antibody-enriched infected milkweed sample. The total length of the MYp-Canadensis genome was 694 kb, which is considerably longer than the 584 kb that was previously reported (Table 1, Fig. 1b). This provides a rough coverage estimate of 550× (500× for ONT; 632× for Illumina considering both length and read numbers). The genome was assembled into seven contigs with an N50 of 388 kb, which is also an improvement over the N50 of 8 kb in the previously reported MYp genome [18]. Annotation of the MYp-Canadensis genome using PGAP revealed the presence of 718 genes in total, including 678 coding sequences, 6 rRNA genes (2 each of 5S, 16S and 23S) and 24 pseudogenes. Both copies of the 16S rRNA-encoding genes were typed at the iPhyClassifier as 16SrIII-F, consistent with the PCR results and with the known classification of MYp as a 16SrIII-F phytoplasma [49]. A plasmid of 4.4 kb was predicted by plasmer. In accordance with this prediction, blast analysis of this contig showed strong similarity (93 and 88% sequence identities, e=0.0) to the annotated plasmids of ‘*Ca*. P. pruni’ strains PR2021 (pPR2021) and lettuce witches’ broom phytoplasma LWB (pLWB-01). Examination of the completeness of all the reported 16SrIII genomes from Table 2 using Busco revealed that the highest completeness score, 97.4%, was observed for the genomes PR2021, MYp-Canadensis, 2A1 and ChTDIII. In contrast, the previously reported MYp assembly had a completeness score of 94.0%, and the lowest score (87.3%) was observed for Vc33 (Table 1). In addition, examination of the newly generated genomes in this study using CheckM2 provided genome completeness of ~94% with <0.6% contamination for both genomes (Table S1). These values are consistent with high-quality, low-contamination assemblies.

### Examination of phytoplasma genomes for GroE chaperonin system

**Fig. 2.**
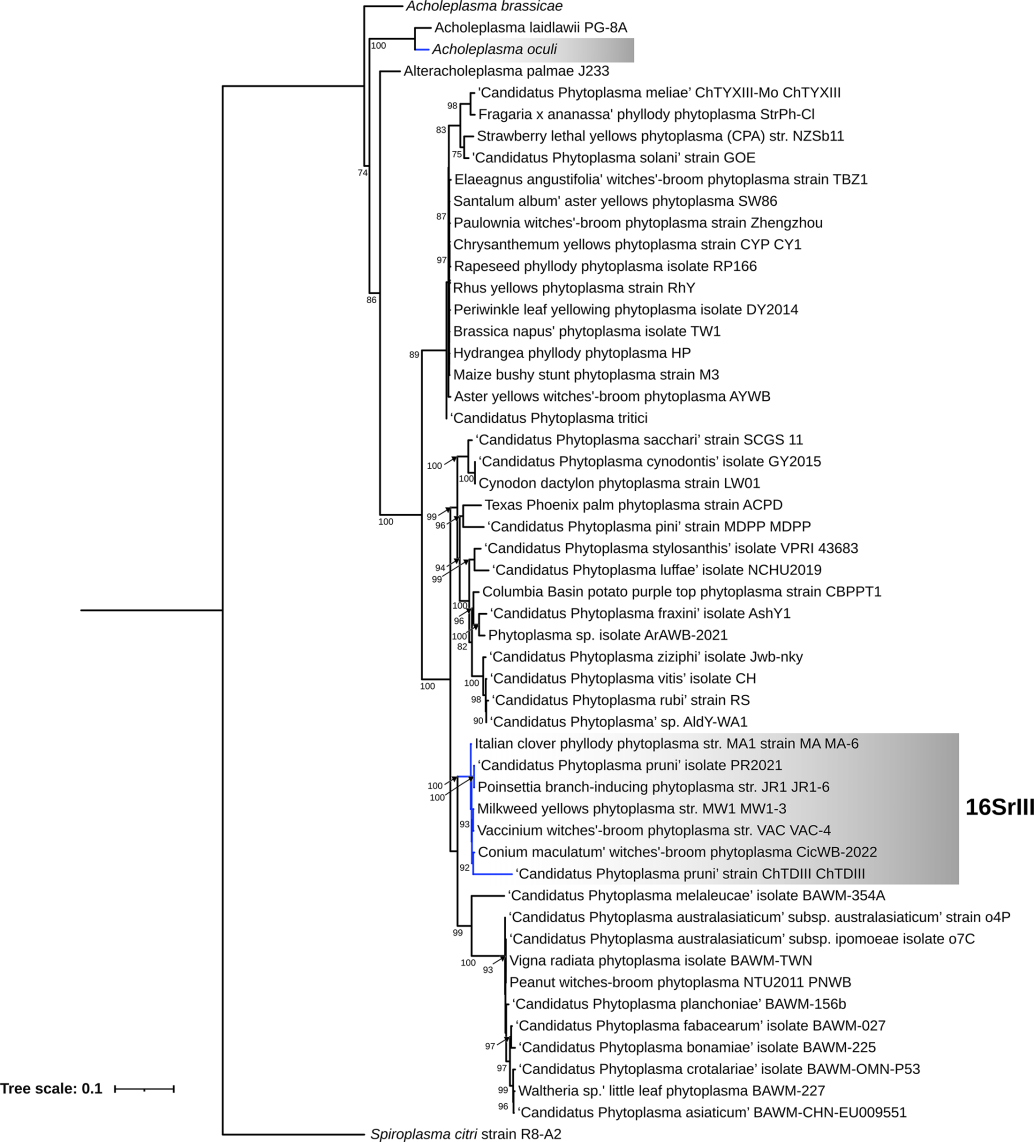
Phylogenetic tree (maximum likelihood; 100 bootstrap replicates) based on 16S rRNA-encoding gene sequences obtained from publicly available genome sequences. Branch colouring is consistent with Schwarz *et al*. [11], with blue branches indicating the 16SrIII taxa that lack the GroE system; in addition, these taxa are highlighted.

In total, 65 unique taxonomic IDs corresponding to a wide variety of phytoplasmas were examined (Table S2). Phylogenetic analysis of the 16S rRNA genes from these phytoplasmas provided results that were consistent with previous 16S gene-based analysis [11,50], with three basal clades represented (Fig. 2). One major group consisted mostly of 16Sr groups I and XII, and this group was more closely related to the *Acholeplasma* taxa compared to the other phytoplasma strains. A second group consisted of a diverse range of phytoplasma taxa, with a complex branching pattern containing six clades representing at least ten ‘*Ca*. Phytoplasma’ species and 6 16Sr groups (IV, V, VII, VIII, XI and XIV). The third major clade consisted of two main branches, including the 16SrIII group (‘*Ca*. P. pruni’), and a second branch consisting primarily of group 16SrII strains, along with ‘*Ca*. P. melaleucae’ (16Sr XXV-A). The 16SrIII group was closely related to the 16SrII group, as reported previously [50].

**Fig. 3.**
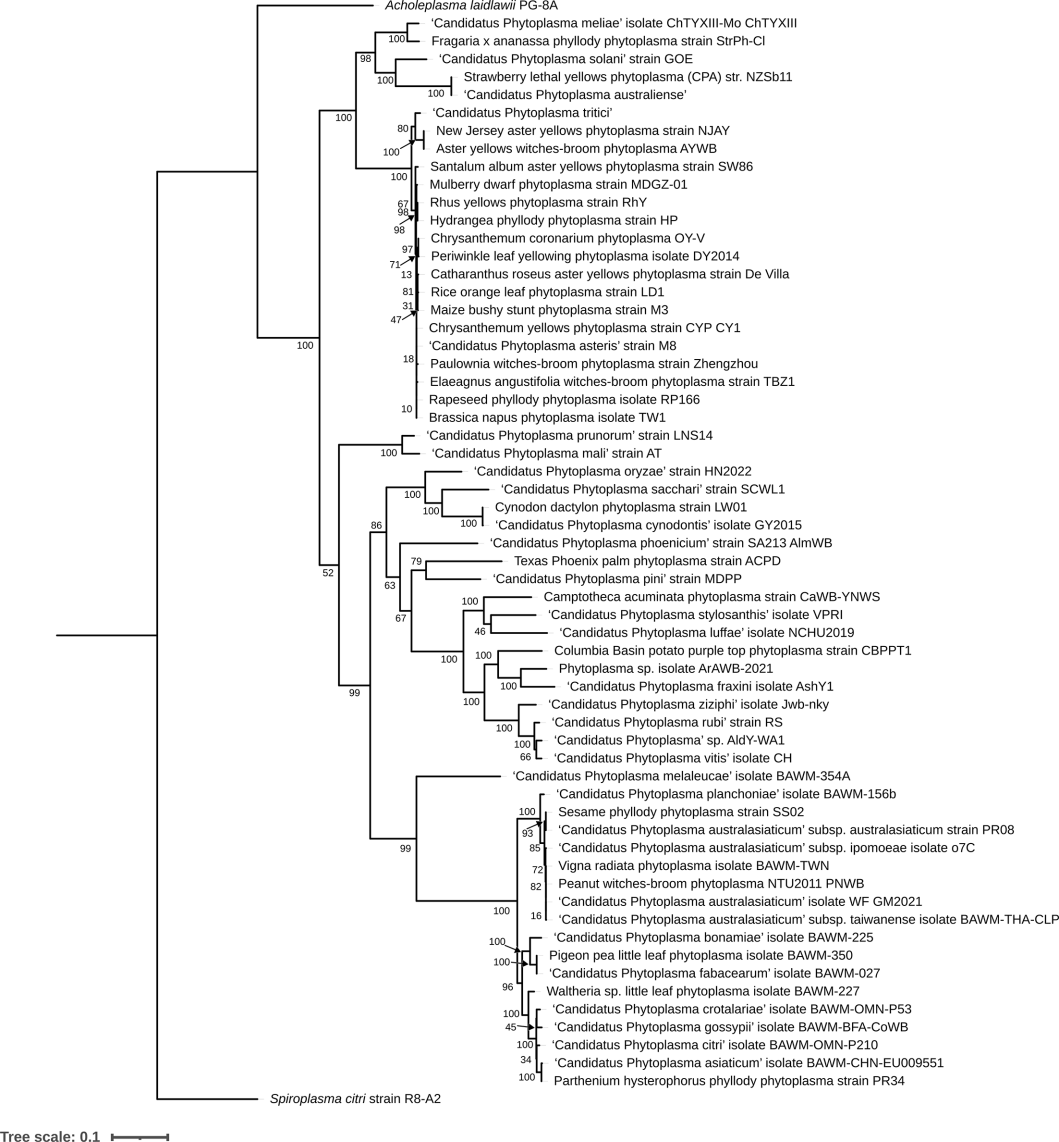
Phylogenetic tree (maximum likelihood; 100 bootstraps) based on full-length *groEL* (*cpn60*) sequences obtained from genome sequences deposited in public databases.

Of the 65 genomes examined, 59 contained an intact GroE system, with both *groEL* and *groES* present in each genome (Table S2). All genomes lacking the GroE system belonged exclusively to the 16SrIII group. In contrast, genomes with intact *groEL/groES* operons represented a broad taxonomic diversity, encompassing 31 phytoplasma species across 16 distinct 16Sr groups (Table S2). These results indicate that, among all high-quality phytoplasma genomes sequenced to date, ‘*Ca*. P. pruni’ (16SrIII) is the only lineage missing the GroE system. In all other phytoplasma groups, *groEL* and *groES* are consistently present and located adjacent to one another within their genomes. While it was previously noted that four phytoplasma genomes from 16SrIII lack a GroE system [18], this observation can now be extended to 12 genomes, 6 of which feature detectable *groEL* pseudogenes. Furthermore, while the GroE system was known to be well represented within phytoplasma groups other than 16SrIII [51,54], it was previously not known if 16SrIII is indeed the only exception. The results shown here provide strong evidence that the GroE system is widely distributed in phytoplasmas other than 16SrIII. Phylogenetic analysis of full-length *groEL* sequences representing all of the phylogenetic diversity that is currently present in public databases (NCBI) is shown in Fig. 3. This analysis demonstrates the very wide sequence divergence of this phylogenetic marker, even in strains that are relatively closely related, underscoring the utility of this marker for differentiation of phytoplasma strains outside of the 16SrIII group [54].

### Pseudogenization of *groEL* in 16SrIII phytoplasmas

tBLASTn analysis of the genomes of all group III phytoplasmas showed that, consistent with the initial report, many of the sequenced genomes from this group contained no coding sequences with any significant similarity to amino acid sequences of phytoplasma GroEL. However, 6 of the 12 genomes did contain nucleotide sequences encoding predicted amino acid sequences that were strikingly similar to GroEL from other phytoplasmas (Table 2). These amino acid sequences were short (50–180 residues) and could not encode a functional protein. One of these putative pseudogenes, encoded on the genome of ‘*Ca*. P. pruni’ strain CX (GCA_001277135.1), was annotated as a pseudogene (labelled ‘hypothetical protein’ by the Prokaryotic Genome Annotation Pipeline at GenBank) [33], while the others were identified by local custom blast databases.

**Fig. 4.**
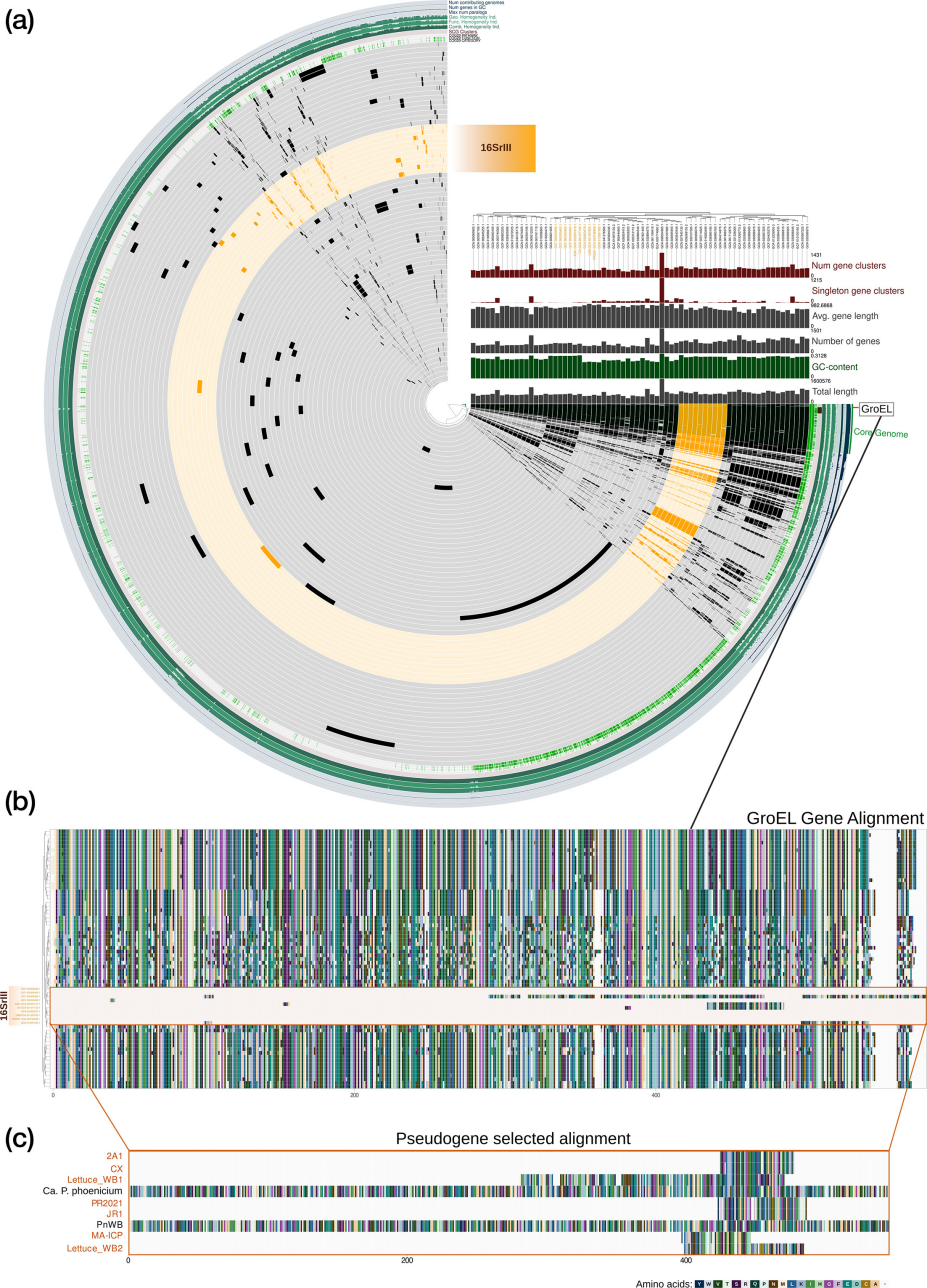
Comparative genomic and phylogenetic analyses of phytoplasma *groEL* genes and pseudogene variants. (**a**) Pangenome matrix and phylogenomic tree of all phytoplasma genomes showing gene cluster distribution across lineages. The outer green ring represents core genes, while the orange-shaded clade highlights members of the 16SrIII group, which lack an intact GroE system. Associated bar plots indicate genomic features, again with orange shading indicating 16SrIII strains, including the total number of gene clusters, singletons, mean gene length, G+C content and total genome size. (**b**) Multiple sequence alignment of GroEL amino acid sequences showing conservation patterns across taxa, with the 16SrIII clade exhibiting high sequence fragmentation and divergence. (**c**) Detailed alignment of representative pseudogenized *groEL* sequences from selected 16SrIII phytoplasmas, illustrating frameshifts, internal stop codons and truncations compared with intact orthologues. A detailed alignment of the predicted amino acid sequences of the pseudogenes and the intact orthologues in the related strains peanut witches’ broom (16SrII) and ‘*Ca*. P. phoenicium’ (16SrIX) is shown in Fig. S1.

A pangenome analysis representing all reported phytoplasma taxa was performed in Anvi’o to explore the distribution and evolutionary fate of *groEL* across group 16SrIII phytoplasmas as a supplementary approach (Fig. 4a). The circular representation highlights the core and accessory genomes, revealing a highly reduced shared gene repertoire and similar gene loss pattern among 16SrIII members. Within this context, the *groEL* cluster is located near the core boundary, but its fragmentation and partial or complete absence in several genomes indicate ongoing pseudogenization (Fig. 4b). Six genomes retained only truncated *groEL*-like sequences of 50–180 amino acids, consistent with previous blast-based detection of pseudogenes (Fig. 4b). The co-occurrence of these remnants within the same syntenic neighbourhood as intact *groEL* orthologues from 16SrII and 16SrIX phytoplasmas further supports a possible single ancestral loss event followed by progressive decay. Together, the Anvi’o pangenome and alignment analyses suggest that *groEL* degradation in 16SrIII phytoplasmas reflects lineage-specific genome reduction rather than multiple independent deletion events.

**Fig. 5.**
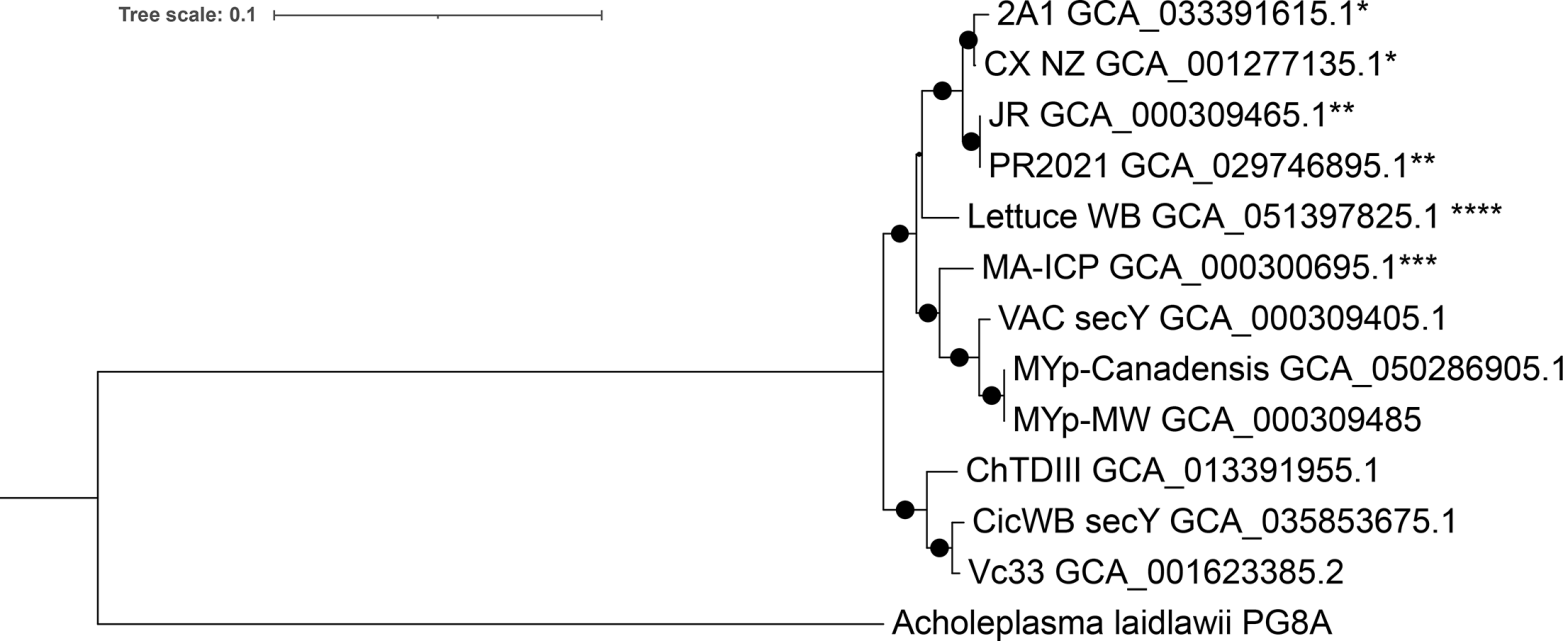
Phylogenetic tree of ‘*Ca*. P. pruni’ (group 16SrIII) based on concatenated taxonomic markers, showing the taxa containing the three different types of pseudogenes. Genomes encoding the types of pseudogenes are indicated by asterisks: type 1–55 amino acids, *; type 2–62 amino acids, **; type 3–50 amino acids, ***; type 4–180 and 87 amino acids, ****.

Alignment of these predicted amino acid sequences to the 546 amino acid sequence of GroEL from PnWB phytoplasma (16SrII, a taxon that is in the same phylogenetic clade as 16SrIII based on 16S rRNA gene sequences – Fig. 2) and ‘*Ca*. P. phoenicium’ (16SrIX, the taxon to which the putative pseudogene amino acid sequences had the most similarity) revealed that the gene fragments encoded amino acids near the carboxy terminus of the protein (Figs 4c and S1). Phylogenetic analysis of the 16SrIII phytoplasma strains based on the sequences of five taxonomic markers (*secY*, *secA*, *nusA*, *tuf* and *rp*) revealed that the four types of identified pseudogenes clustered consistently with the phylogenetic placement of the phytoplasmas containing them (Fig. 5). The putative pseudogenes encoding these amino acids are considered to be unitary pseudogenes, in that there is no evidence of gene duplication in the genome, and the loss of these genes is associated with a complete loss of function [55]. No evidence of nucleic acid sequences encoding amino acids with similarity to GroES was found in any of the genomes.

**Fig. 6.**
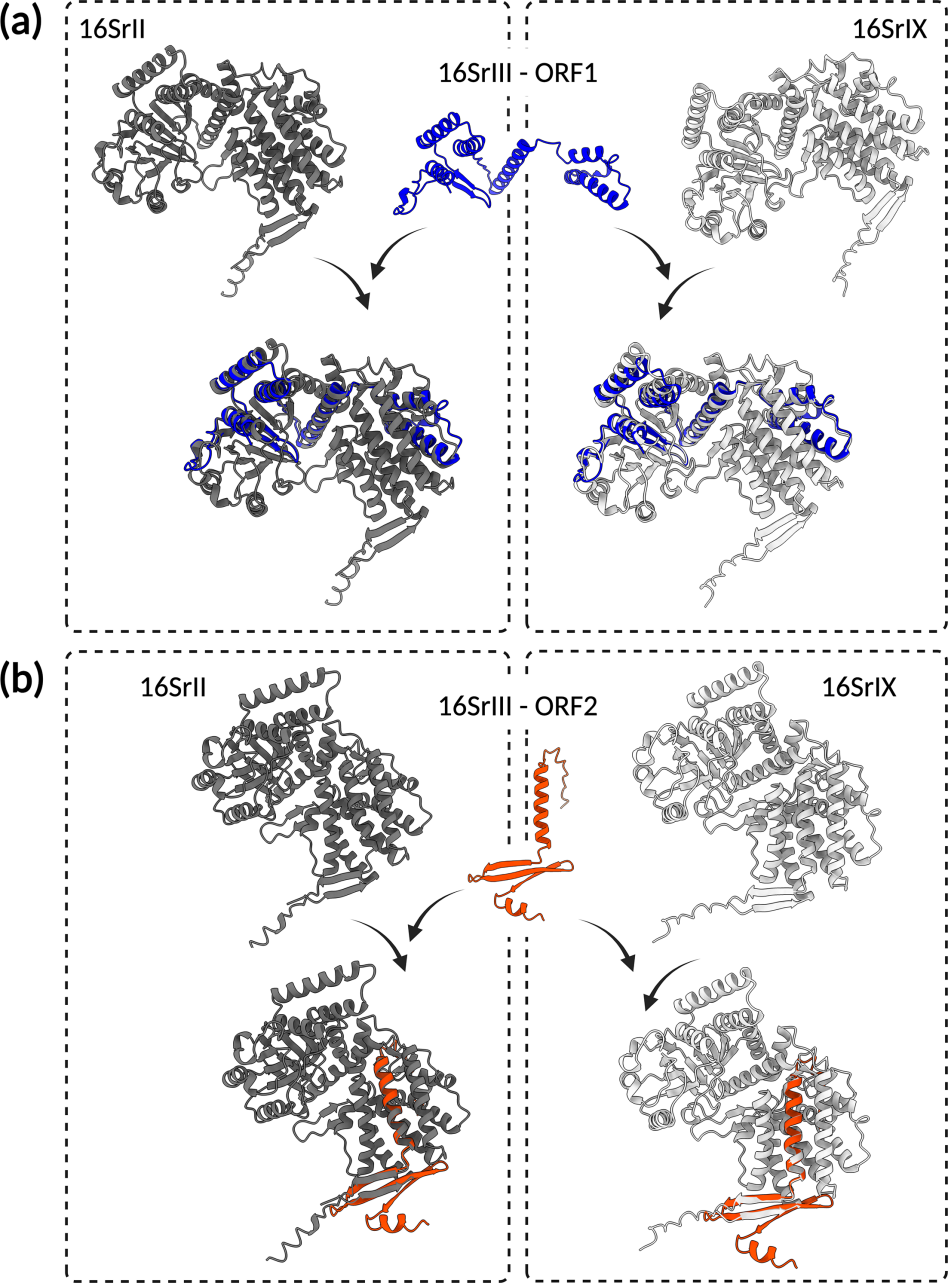
Structural modelling of GroEL proteins from 16SrII, 16SrIX and the predicted proteins from putative GroEL pseudogenes of lettuce witches’ broom phytoplasma. ORF1, 180 amino acids; ORF2, 87 amino acids. Animations of these models are provided as supplementary files.

Structural models were compared for the GroEL proteins from peanut witches’ broom phytoplasma strain NTU2011 (16SrII), ‘*Candidatus* Phytoplasma phoenicium’ (16SrIX) and the two predicted pseudogenes from lettuce witches’ broom phytoplasma (16SrIII, which had the longest predicted proteins of 180 and 87 amino acids). Substantial overlap of the predicted protein structures was observed towards the carboxy terminus of the intact proteins, consistent with the amino acid sequence alignment (Figs 6 and S1). ORF1 (180 amino acids) retained partial apical, intermediate and equatorial domains, while the smaller ORF2 (87 amino acids) retained only a partial equatorial domain (Fig. 6). Neither of these protein fragments is likely sufficient to support multimerization based on models established from crystal structures of *Mycobacterium tuberculosis* Cpn60.2 [56]; however, no crystal structures have been reported for phytoplasma GroEL proteins.

To test whether relaxed purifying selection preceded *groEL* degradation, we performed comparative dN/dS analysis using the RELAX framework [42]. We compared 133 intact *groEL* orthologues from diverse phytoplasma groups against 13 fragmented *groEL* remnants from '*Ca*. P. pruni' (16SrIII).

Structural analysis revealed severe degradation in 16SrIII fragments (mean length 204 bp, 95% CI: 118–289) compared to intact orthologues (mean length 1,437 bp, 95% CI: 1,362–1,513), representing an 85.8% size reduction (*P*=5.65×10⁻¹⁸, Table 3). Additionally, 31% of fragments contained internal stop codons, compared to 0% in intact sequences.

**Table 3.** Structural and molecular evidence for *groEL* pseudogenization in 16SrIII

Parameter	Intact (*n*=133)	Fragmented (*n*=13)	***P*-value**
Mean length, bp (95% CI)	1,437 (1,362–1,513)	204 (118–289)	<10⁻¹⁷
Sequences with internal stops (%)	0 (0%)	4 (31%)	<0.001
Usable for dN/dS analysis (%)	132 (99%)	1 (8%)	<10⁻¹⁵
RELAX k parameter	Reference	0.92*	1.0†

*Point estimate; CI not available due to boundary conditions.

†Likelihood ratio test; underpowered due to class imbalance.

The advanced state of pseudogenization constrained molecular evolution analysis, as only 1 of 13 fragments retained sufficient structural integrity for codon-level alignment and dN/dS calculation. While the RELAX relaxation parameter (k=0.92) suggested a trend towards relaxed selection in fragmented sequences, the analysis was underpowered (*P*=1.0). Notably, this severe structural degradation indicates that *groEL* pseudogenization in 16SrIII is an ancient event, with selection fully relaxed over an extended but undetermined evolutionary period. The extent of degradation precludes robust molecular clock analysis and is consistent with irreversible gene loss rather than recent functional decay.

### Conserved synteny of the GroE system in 16SrII and pseudogenes of 16SrIII

To determine if the pseudogenes were orthologous by synteny, rather than simply a case of coincidental similarity [55], the gene neighbourhood of the intact GroE system in two close phylogenetic neighbours of 16SrIII, ‘*Ca.* P. aurantifolia’ (PnWB; 16SrII) and ‘*Ca.* P. phoenicium’ (SA213; 16SrIX), was examined and compared to the locations of the putative pseudogenes.

**Fig. 7.**
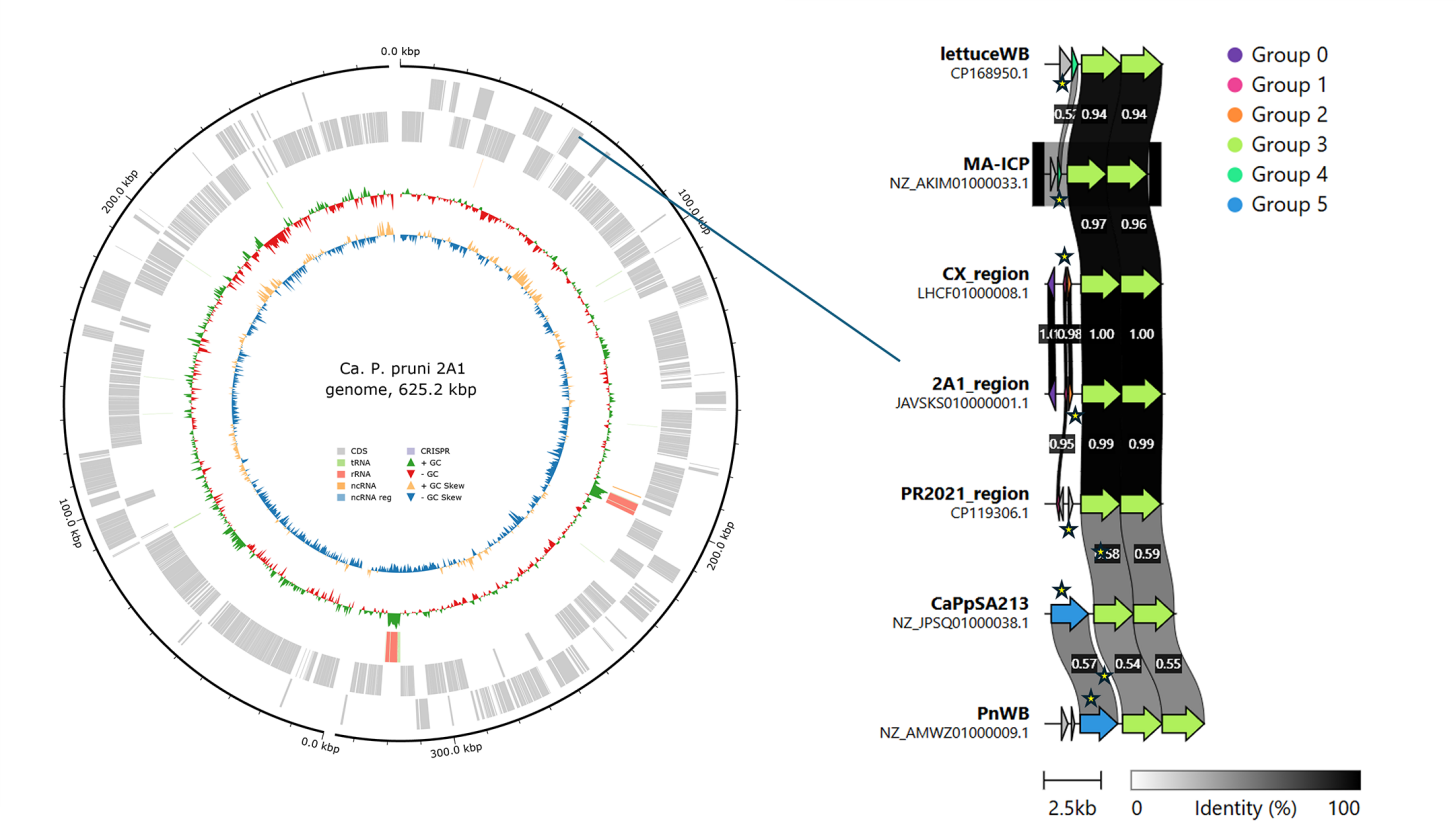
Gene locations of the predicted pseudogenes and the adjacent genes in 16SrIII and related strains. The location of the gene cluster in the genome of strain 2A1 is indicated by a line. Genes annotated by Prokka as *groEL* (*cpn60*) are indicated by a star. Groups of genes sharing high similarity scores are indicated by colouring. Amino acid similarity scores are indicated between adjacent strains that are connected by coloured blocks, with darker colours indicating higher scores. The full matrix of amino acid similarity scores for all gene combinations is shown in Table S3.

The GroE system (*groEL*/*groES*) in PnWB is found on contig 9 (reverse strand) and is immediately adjacent to two genes that are both annotated as ‘multidrug resistance ABC transporter ATP-binding and permease protein’ (Fig. 7). These genes are identified as *evbH* (contig 9 : 24713–26554, reverse strand) and *evbG* (contig 9 : 26551–28284, reverse strand). Considering the transcriptional orientation, the gene order in this part of the PnWB genome is *groEL-groES-evbG-evbH*.

In the 16SrIII phytoplasmas that contain putative *groEL* pseudogenes, there are two genes located immediately next to each pseudogene (Fig. 6). These protein-coding genes are annotated variously as ‘multidrug resistance ABC transporter ATP-binding and permease protein’ (strain PR2021), or ‘ABC transporter ATP-binding protein’ (strains 2A1, CX and MA-ICP). These genes encode proteins that are similar in size to PnWB *evbG* and *evbH* – 576 out of 577 amino acids for *evbG* and 593 out of 613 amino acids for *evbH* (Table 2). In each 16SrIII genome, the putative pseudogene is immediately adjacent (separated by 159–429 nucleotides) to a gene that is similar in size to *evbG* of PnWB. This conserved proximity suggests that the putative pseudogene in 16SrIII is found in a genomic context that is similar to that of the intact *groEL* gene in PnWB (Table 2; Fig. 7).

To determine if these similar genes are likely orthologues, we compared the predicted amino acid sequence similarity of these genes in PnWB and the 16SrIII strains. Within the 16SrIII genomes, the two genes immediately downstream of the putative *groEL* pseudogenes had amino acid similarity scores (by clustalw) of 94–100%, suggesting that they are orthologous genes within these strains (Fig. 5, Table S3). The amino acid similarity scores of these 16SrIII genes to the PnWB *evbG* and *evbH* were 54 and 55% (Fig. 6, Table S3). These scores are similar to the amino acid similarities of the orthologous taxonomic markers *secY*, *secA*, *nusA* and *tuf* in the 16SrII and 16SrIII genomes, which ranged from 38 to 56% (Table S4). In contrast, when all other genes in the PnWB chromosome with annotations containing the phrase ‘ABC transporter ATP-binding’ were compared to the genes immediately upstream of the putative *groEL* pseudogenes in 16SrIII, the amino acid similarity scores were much lower – 15–20% (data not shown). These amino acid sequence similarity comparisons suggest that the two genes encoding ABC transporters in 16SrIII are indeed orthologues of *evbG* and *evbH* in PnWB, confirming the syntenic relationship of *groEL* genes and pseudogenes in these closely related phytoplasmas.

We also determined the syntenic relationships of annotated genes across a wider genomic window (Fig. S3). This analysis confirmed the strong similarity of genome structures in the regions containing the *groEL* pseudogene and the *evbG/evbH* putative orthologues, and none contained any evidence of a *groES* gene remnant. To provide an alternative visualization of the genomic regions, we examined the automated annotations at JGI-GOLD. Only two of the 16SrIII strains are publicly available as annotations at JGI-GOLD, and the annotations in this area of each genome were compared to the closest phylogenetic relative, PnWB NTU2011. The genomic region upstream of the *groEL* pseudogenes in 16SrIII strain CX contained no evidence of the shorter *groES* genes and displayed a dearth of annotated genes in general compared to the corresponding gene region in PnWB (Fig. S2). Only a few short hypothetical proteins were annotated in the strain CX genome, on the opposite strand of the *groEL* pseudogenes and *evbG*/*evbH* (Fig. S2). The reason for this genomic ‘desert’ in strain CX is unclear, but this area seems to have been subjected to an ancient pseudogenization that resulted in the loss of the GroE system, with only a small vestige of the *groEL* gene remaining in this region in certain extant strains. This was not the case for the 16SrIII strain 2A1, but there was no evidence of a gene with any similarity to *groES* in the area downstream of the *groEL* pseudogene (Fig. S2).

### No evidence of recent *groEL* re-acquisition in non-fragmented 16SrIII genomes

Due to the clear cases of *groEL* acquisition by HGT that were previously documented in *Mollicutes* [11,12], we examined 16SrIII genomes for the presence of any *groEL* genes, even those more distantly related to phytoplasmas. No evidence of intact genes with any similarity to *groEL* was observed in any of the genomes. Many of the previously reported 16SrIII genomes are highly fragmented (more than 150 contigs) based on short-read but deep and accurate sequencing chemistry (Illumina). In addition, the assembly process commonly involves a mapping step to exclude non-phytoplasma DNA because of the metagenomic nature of phytoplasma DNA samples. To address this possible source of error, we prepared an MYp assembly by only removing host reads (chromosomal, mitochondrial and chloroplast), leaving all non-host reads (mostly bacterial, but possibly also including other host-associated micro-organisms) available for assembly. Removal of host reads left 21,754,990 out of 57,879,811 Illumina reads (37.6%), compared to 12,482,861 reads (21.6%) that remained when mapped to the previously reported MYp genome sequence. This suggests that many other micro-organisms were associated with the milkweed leaf sample. When these host-depleted, assembled reads were queried for the presence of *groEL*-like sequences, 9 sequences were retrieved, all from bacteria (Table S5). Examination of the assembled contigs containing these *groEL* sequences (the chromosomal context) provided taxonomic identifications consistent with the *groEL* sequences in all cases (Table S5). These results exclude the possibility of a *groEL* gene being acquired by HGT, as its sequence would then be located within a context of phytoplasma-like DNA sequences.

## Conclusions and implications

Here, we have provided two additional high-quality, non-fragmented assemblies of 16SrIII phytoplasmas, and we observed no evidence of the presence of a *groEL* gene encoding a protein that could be functional. In addition, no evidence of recent re-acquisition of *groEL* was observed in the genomes reported here or in previously published assemblies that are less fragmented. All other phytoplasma genomes examined, representing a very wide taxonomic diversity of these micro-organisms, possess genes encoding evidently full-length GroES and GroEL proteins. Therefore, we conclude that 16SrIII genomes are unique among phytoplasmas in that they contain no functional GroE system.

Given that all bacterial taxa except most of the *Mollicutes* possess a functional GroE system, this is thought to be the ancestral state for bacterial taxa. Among Mollicutes, GroE is present only in select groups, which include the basal clade *Acholeplasmataceae*. Through genome sequencing and analysis of previously sequenced genomes, we have demonstrated that group 16SrIII phytoplasmas have lost the ancestral GroE system, which is retained in all other taxa of phytoplasmas. No evidence of the presence of *groEL/groES* genes was observed, indicating that these bacteria, like most other *Mollicutes*, have not re-acquired the functionality of GroE. Since *groEL* is absent in an obviously pathogenic group of phytoplasma, it seems unlikely that the protein is involved in pathogenesis as has been speculated from observations in other *Mollicutes* [12], at least for the phytoplasmas within group 16SrIII. While GroEL may play a role in pathogenicity in other phytoplasmas, for this group, the evidence presented indicates that the GroE system is dispensable for pathogenicity. These observations suggest that the protein most likely retains a canonical role in the folding of host cell proteins. Those *Mollicutes* that have lost the ancestral genes during genome reduction seem to have crossed the low evolutionary barrier identified by Schwarz *et al.* [11] and adapted their client proteins to be able to fold in the absence of this system.

## Abbreviations

HGT: horizontal gene transfer
iTOL: Interactive Tree of Life
